# Supplementary figures and images for: easyXpress: An R package to analyze and visualize high-throughput C. elegans microscopy data generated using CellProfiler
Source: PLoS One. 2021 Aug 12;16(8):e0252000. doi: 10.1371/journal.pone.0252000 (PMC8360505; doi:10.1371/journal.pone.0252000)

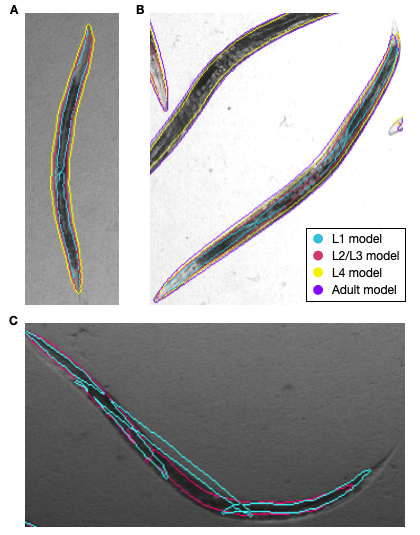

Supplement: S1 Fig — When running CellProfiler’s WormToolbox with multiple worm models, multiple model objects can be assigned to a single primary object (real animal). Different colors are used to outline each worm model object. Here four unique models were used: L1, L2/L3, L4, and Adult. These worm models represent the approximate sizes of animals at each life stage. For example, some mutant or diverse wild genetic backgrounds might have differently sized adult animals as compared to the laboratory-adapted N2 strain. We have included this “soft matching” to account for small differences in the sizes of developmental stages across different genetic backgrounds, laboratories, and environmental conditions. (A) An animal detected by CellProfiler as a primary object has been assigned three unique worm models: two L1 model objects, one L2/L3 model object, and one L4 model object. modelSelection() classifies this animal as an L4 model object. (B) An animal detected as a primary object has been assigned four unique worm models: three L1 model objects, two L2/L3 model objects, one L4 model object, and one Adult model object. Here, modelSelection() identifies the Adult model as the best fitting model object. (C) An animal detected as a primary object has been assigned two unique worm models: three L1 model objects, and two L2/L3 model objects. In this case, modelSelection() classifies this animal as an L2/L3 model object and adds a cluster flag annotation to indicate the repeated assignment of the selected model object to the primary object. (TIFF) [file pone.0252000.s001.TIFF]

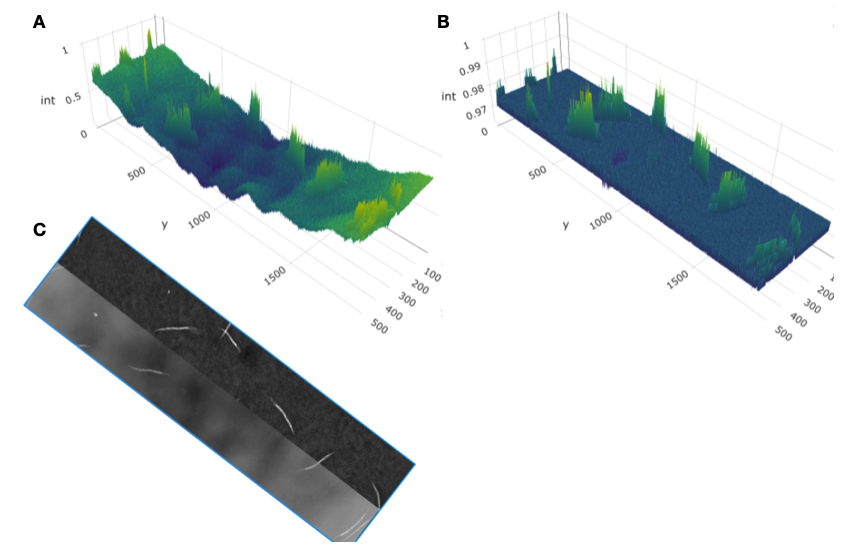

Supplement: S2 Fig — (A) Left is raw intensity values across well. (B) Right is with background correction. Intensities of object illumination are displayed on each z-axis. Objects near the edge of the well (y < 500 and y > 1500) have similar raw detected intensities (int) to more medial objects (y ~ 1000) in (A) but lower corrected intensities in (B) because of uneven background correction. Raw and background-corrected image segments are displayed in (C). Notice animals on the edges of the well do not stand out from the background as much as animals in the center of the well and therefore are more challenging to discern. (TIFF) [file pone.0252000.s002.TIFF]

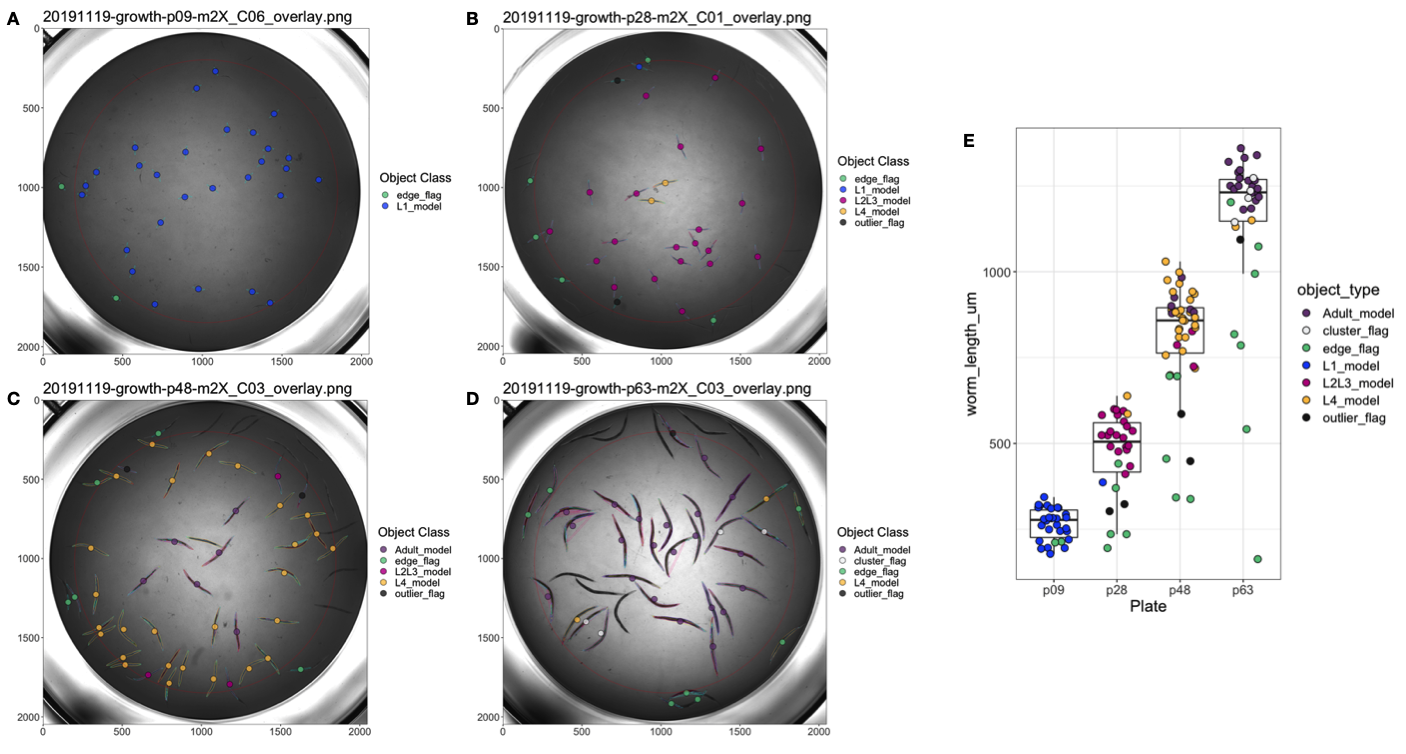

Supplement: S3 Fig — Similar to Fig 5, well images taken at (A) 9 hours indicating the L1 stage, (B) 28 hours indicating the L2/L3 stage, (C) 46 hours indicating the L4 stage, and (D) 63 hours were analyzed. Here, the raw data results are displayed. The length of each identified object identified is shown in (E). (TIFF) [file pone.0252000.s003.TIFF]
