# Supplementary material for: easyXpress: An R package to analyze and visualize high-throughput C. elegans microscopy data generated using CellProfiler
Source: PLoS One. 2021 Aug 12;16(8):e0252000. doi: 10.1371/journal.pone.0252000 (PMC8360505; doi:10.1371/journal.pone.0252000)
Supplement: Attachment — Submitted filename: Response to Reviewers.pdf [file pone.0252000.s004.pdf]

Dear Heidi,

We appreciate the care that the reviewers took in evaluating our manuscript. We believe that the manuscript is certainly improved after these suggestions. We have indicated our responses to reviewers' comments below.

Reviewer #1:

Authors has developed a package easyXpress based on R-Statistical Environment platform that can facilitate the analysis with CellProfiler. This study has potential and could be beneficiary for many scientists looking for a solution. However, I find it difficult to assess the methods that they have developed due to limitation posed with unavailability of the code as well as statistical description of functions. In addition, I have few questions to authors as follows.

Q.1 : Authors mentioned that the code is available on github, however, I was unable to see the code or validate that it is working in the way it is proposed.

The complete source code, example data, and extensive documentation are publicly available on GitHub: https://github.com/AndersenLab/easyXpress. A user may freely browse the code for each function at: https://github.com/AndersenLab/easyXpress/tree/master/R. Alternatively, we have provided a publicly available tutorial on the usage of easyXpress at: https://rpubs.com/jnyaanga/765641.
If the reviewer wishes to download all source code to their local machine for closer examination, they may also use Git to clone the full repository: https://github.com/AndersenLab/easyXpress.git.

All of these data and code were available at the time of submission. Additionally, several users (from the international worm meeting) have downloaded and used the package. Therefore, we know that it is available.

We are confused about why the reviewer could not access our repositories when others could. Please offer us more specificity and we can look into the potential issues.

Q.2 Many place it is written to refer the manual however manual is not accessible, and information is not provided in the manuscript. My suggestion is, since it is package -based article, authors must provide the code to validate their claim or write full description in the manuscript.

Again, all source code and documentation are publicly available on GitHub. A GitHub account is not required to readily view the repository. Simply visit: https://github.com/AndersenLab/easyXpress. As we stated above, a vignette is available as often used by many R package developers. Other researchers have used these repositories and followed our vignette. This issue seems to be specific to the reviewer.

Q.3 Authors mention that they have developed some functions to customize the filtering of noise in data, however no description or statistics underlying the function is explained anywhere in manuscript.

The following functions are involved in filtering data: *edgeFlag()* and *setFlags()*.

In the main text, we described that *edgeFlag()* sets the radius of a circular well to 825 pixels to better identify animals located beyond this radius. This radius value is easily adjusted by the user to serve project-specific analyses. No statistics are described because none are needed. It is a user-defined filter value.

In the main text, we also explained the purpose of *setFlags()* to detect outlier measurements among all measurements within a well using Tukey's fences.

Without explicit knowledge as to which function the reviewer is referring, we are unable to provide additional statistical descriptions. No other functions deal with noise in the data. Please let us know what specific functions are of concern.

Q.4 I am unable to find the explanation/statistics for summarization in methods though it is mention.

We developed the function *process()* to aid in the summarization of data. In the main text, we state: "This function comprehensively calculates the means, variances, quantiles, minimum, and maximum values of animal length for any experimental unit (*e.g.* well)."

We are unclear what further explanation the reviewer would need because each of these statistical summaries are a part of any standard analysis. Please let us know what specific explanations or summarizations are needed.

Reviewer #2:

This paper presents a piece of software meant to aid in the management and analysis of data first examined by CellProfiler. Beyond offering a method to visualize the outputs of CellProfiler, with the option of overlaying annotations, it provides further functionality in terms of refining annotations and removal of C. elegans deemed as outliers by custom filtering routines. Flexible control of functions and hyperparameters make this software amenable to an array of imaging situations. The contributions of this software, although limited when compared to a package such as CellProfiler itself, seem meaningful as it automates processes that are otherwise menial and time consuming. Further, inter-experimental comparisons will benefit from the consistency offered by using this software to manage how outlier removal occurs within a dataset. This paper makes meaningful contributions to the field of study however, there remains a few outstanding questions after reading the paper. Those questions can be found below.

Lines 130-143 begin to describe the modelSelection function, however, some items remain unclear. Based on the description starting at line 136, it seems to be that modelSelection would select the most infrequent model as the best model fit. Given that this is the correct interpretation, it seems to run against intuition where if one were to rely on model frequency alone to clear up cases of ambiguous labeling, the most frequent model may be better suited. If the least frequent is indeed the model that is chosen in cases of ambiguity, it would be helpful to elaborate on why this is the case. If this was understood incorrectly, it would be helpful to expand upon this sentence and provide further details on how models are ranked/selected.

We completely agree that the *modelSelection()* step could use additional clarification. In the main text, we have added the following:

- In instances where multiple worm model objects are assigned to a single primary object, *modelSelection()* will identify the best fitting model. Models are first ranked by frequency in the dataset

such that the smallest model is classified as the most frequently occurring and the largest model is the least frequently occurring. In our experience, the most frequently occurring model in the dataset has the smallest size because it is often repeatedly assigned to a single primary object. Conversely, the least frequently occurring model in the dataset has the largest size as it is able to define the entire size of an animal and is thus assigned to a primary object only once. The largest ranked model occuring within a single primary object is then selected as the best fitting model for that primary object.

The following sentence starting on line 139 raised another question. It seemed to be in the previous sentences that a "primary object" was referencing a C. elegans however, the statement "modelSelection() will also specify whether the selected model object was repeatedly assigned to the same primary object..." makes this conclusion seem unlikely. It is understood that a single C. elegans could be assigned multiple instances of different models however, it is unclear how a C. elegans could be assigned multiple instances of the exact same model. Due to this confusion, it would be helpful to have a statement on either i) what a primary object is in reference to, if it is not a C. elegans, or ii) in what cases one would expect a C. elegans to receive duplicates of the same model label.

We agree this section could use further clarification. We added S1C Figure to provide a visual example for how a selected model object can be repeatedly assigned to the same primary object. We have clarified this point in the text with the following addition:

- The largest ranked model is then selected as the best fitting model. If necessary, *modelSelection()* will also specify whether the selected model object was repeatedly assigned to the same primary object and flag this event as a cluster. This problem occurs in instances where a model object is repeatedly assigned to a single primary object. If the largest model object is found to occur repeatedly in a single primary object, this model will be selected and a cluster flag will be added (S1C Fig).

The authors indicate that the easyXpress will help with necessary statistical tools for quality control and provide quantitative analysis of high-throughput imaging datasets. Although Figure 4 indicates a study using paraquat on worm measurements, the authors did not provide many details neither present quantitative results from this data. It is advisable to indicate the experimental details in the method section and show a detailed statistical analysis of this data. To show the full power of this platform, the author should represent the quantitative data of the study, worm body sizes, and the necessary statistical analysis.

We thank the reviewer for their request for clarification and inclusion of these data. We have added the necessary experimental details to the Methods section:

- Paraquat dose response
  A 1.5 M solution of paraquat (Methyl viologen dichloride, Sigma, 856177-1G) was prepared in sterile water, aliquoted, and frozen at -20°C until used. Experimental animals were grown at 20°C and fed OP50 bacteria spotted on modified nematode growth medium, containing 1% agar and 0.7% agarose to prevent animals from burrowing. After three generations of passaging, animals were bleach-synchronized and embryos were transferred to the wells of 96-well microplates. Each well contained approximately 30 embryos in 50 µL of K medium (Boyd et al. 2012). Microplates were incubated overnight at 20°C with continuous shaking. The following day, arrested L1 animals were fed HB101 bacteria suspended in K medium. At the time of feeding, the animals were also exposed to paraquat at one of six concentrations (0, 7.81, 31.25, 125, 500, 2000 µM) by serial dilution of a freshly

thawed aliquot of 1.5 M paraquat solution. The final volume in each well after dosing and feeding was 75 µL. The animals were then grown for 48 hours at 20°C with continuous shaking, afterwards the microplates were imaged to assess the effects of paraquat exposure on nematode development.

We have also revised Fig. 4 to include a panel displaying the length of animals over the dose range.

Figure 3 identifies a few animals as L4_model while they appear to be of similar size as the adult animals as per the box plot on the right. Were these animal images confirmed with high-resolution imaging or manual inspection? If these annotations were erroneous, the authors are encouraged to discuss possible reasons for this error and whether it will affect the measurements.

Thank you for this comment because it allows us to clarify a significant source of confusion. These annotations were not erroneous. In order to fully capture the range of animal sizes throughout development, we include overlapping animals when building a model training set. In this way, the L4 model is trained on a set of images that include early Adult animals, and ultimately might detect animals that appear similar to adults in size. However, we want to clarify that our models do not designate stage assignments. This "soft matching" is merely a way of capturing the vast differences in animal size that occur during *C. elegans* development, especially when strains different from the laboratory-adapted N2 strain are scored. The labeling of a primary object as an L4 model object does not designate that the animal is developmentally within the L4 stage, simply that they fit within the parameters defined by the L4 model training set.

To better clarify this concern in the text, we have added the following:

- During the implementation of easyXpress, four unique worm models representing *C. elegans* life stages were calibrated and applied: L1, L2/L3, L4, and Adult. These worm models do not designate stage assignments but rather represent the approximate sizes of animals that fall within the respective age groups.

In S1 Fig, we have included additional information for the purpose of the "soft matching":

- For example, some mutant or diverse wild genetic backgrounds might have differently sized adult animals as compared to the laboratory-adapted N2 strain. We have included this "soft matching" to account for small differences in the sizes of developmental stages across different genetic backgrounds, laboratories, and environmental conditions.

We look forward to receiving your response to our revisions.

Joy Nyaanga
Timothy A. Crombie
Samuel J. Widmayer
Erik C. Andersen
